# Short-term plant-community responses to large mammalian herbivore exclusion in a rewilded Javan savanna

**DOI:** 10.1371/journal.pone.0255056

**Published:** 2021-07-22

**Authors:** Arjun B. Potter, Muhammad Ali Imron, Satyawan Pudyatmoko, Matthew C. Hutchinson

**Affiliations:** 1 Department of Ecology and Evolutionary Biology, Princeton University, Princeton, New Jersey, United States of America; 2 Wildlife Laboratory, Faculty of Forestry, Universitas Gadjah Mada, Yogyakarta, Indonesia; Estacion Experimental de Zonas Aridas, SPAIN

## Abstract

Grassy biomes such as savannas are maintained by an interacting suite of ecosystem processes from herbivory to rainfall to fire. Many studies have examined the impacts of large mammalian herbivores on herbaceous plant communities, but few of these studies have been conducted in humid, fertile savannas. We present the findings of a short-term experiment that investigated the effects of herbivory in a fertile, humid, and semi-managed savanna. We erected large-herbivore exclosures in Alas Purwo National Park, Java, Indonesia where rainfall is high and fire is suppressed to test how herbivores impact plant community development across the growing season. Where large mammalian herbivores were excluded, herbaceous plant communities contained more non-grasses and were less similar; diverging in their composition as the growing season progressed. Effects of herbivore exclusion on plant species richness, evenness, and biomass per quadrat were generally weak. Notably, however, two weedy plant species (one native, *Imperata cylindrica* and one introduced, *Senna* cf. *tora*) appeared to benefit most from herbivore release. Our results suggest that heavy grazing pressure by native large mammalian herbivores controlled the composition of the herbaceous plant community. Moreover, exclusion of large mammalian herbivores led to divergence in the plant species composition of exclosures; compositional dissimilarity between herbivore-exclusion plots was higher than between plots exposed to large mammalian herbivores. Our findings suggest that, at this high-rainfall site, large mammalian herbivores constrained the developmental trajectory of plant communities across the growing season.

## Introduction

The next decade has been designated the United Nations Decade for Ecosystem Restoration, and a central aspect of restoration is the rewilding of large herbivore assemblages [1]. Large-herbivores can exert strong forces on plant community structure [2–5], which makes their rewilding crucial to landscape scale restoration efforts. Through selective consumption of plant species and tissues, as well as trampling, defecating, and urinating, large mammalian herbivores alter the growth, colonization, and extinction rates of plants [6]. Moreover, herbivore-induced shifts in plant communities can have important impacts on wider ecosystem processes from disease transmission [7] to desertification [8].

Selective herbivory, caused by variation in the acceptability of plants as food (palatability), is commonly invoked to explain herbivore-induced changes in plant communities. For instance, bison (*Bison bison bison*) suppress the dominant, palatable grass *Andropogon gerardii*, leading to increased forb abundance [9, 10] and, more generally, even low herbivore densities can be sufficient to suppress palatable plants [11]. In addition, herbivory (and rewilded herbivore assemblages in particular) may provide management benefits through the consumption of palatable, invasive plant species [12]. However, herbivores may also facilitate invasive species; in rangelands, selective grazing by cattle is frequently blamed for facilitating noxious invasive weeds [13]. While the directionality of herbivore impacts on plant communities ultimately depends upon the particular herbivore and plant species involved, it is clear that herbivores’ feeding preferences can direct plant community shifts [14]. In addition, indirect and non-consumptive effects of herbivory may have counterintuitive impacts on plant communities. For instance, herbivory may suppress unpalatable species by driving soil quality declines [15] or facilitate the growth of palatable species by stimulating compensatory regrowth and activating plant meristems [16, 17].

The specific conditions of a site—in particular, rainfall, soil fertility, and grazing intensity—are thought to explain much of the variability in plant community responses to large mammal herbivory [6, 18]. However, these relationships are often complex. For example, herbivory is expected to reduce plant species diversity under low productivity conditions and increase diversity at high productivity [19, 20]; yet the largest synthesis of herbivore-exclusion experiments found no significant relationship between herbivore-induced shifts in richness or evenness and temperature, precipitation, or primary production [5]. In principle, higher soil moisture should amplify above-ground competition in herbaceous communities; herbivores, by suppressing competitive dominants, therefore alleviate light competition and may promote diversity [6, 19]. Yet our understanding of how plant communities respond to herbivory at high-rainfall sites remains limited.

There is, however, reason to expect that herbivores in higher productivity sites will have large impacts on plant composition even if their impacts on diversity are minimal. For instance, when large mammalian herbivores were excluded from areas of Kruger National Park (South Africa) plant communities with and without herbivores had similar levels of diversity yet distinct compositions [21]. Similarly, a study showing increases in plant diversity under herbivory also found that plant compositional dissimilarity was higher at high productivity sites [19]. Herbivory-induced compositional shifts may be more apparent at high productivity sites due to both increased competition for light when herbivory is absent as well as rapid grass regrowth and the formation of grazing lawns (where only highly grazing-tolerant plant species persist) when grazing pressure is high [22]. The possibility of these alternative states accords with the expectation that, at higher productivity sites, plant-species richness follows a hump-shaped distribution with grazing intensity, due to stronger divergence between grazing-tolerant and canopy-dominant strategies [18].

While a large body of literature describes the impacts of herbivory in higher rainfall or higher productivity sites, these generally consider mesic environments (500–1000 mm of mean annual precipitation; [12, 19, 20, 23]). Comparatively little is known from savannas that combine >1000 mm annual precipitation and high soil fertility; this extreme may serve as a crucial test of existing knowledge given that both savanna and forest could exist under these conditions [24]. High soil fertility should exacerbate the effects of high rainfall, since fertility facilitates biomass accumulation, resulting in colonization limitation [25]. High fertility should also facilitate heavy grazing, as plants have higher nutrient concentrations but also invest more in growth over defense [26]. Overall, it might be expected that at extremely productive sites, the effects of large mammal herbivory would be qualitatively similar to more mesic sites but larger in magnitude and rapidly evident.

We investigated how herbaceous plant communities developed across a growing season following herbivore exclusion in a semi-managed savanna in eastern Java, Indonesia, which falls at the extreme of the productivity gradient and represents a major geographical gap in herbivore-exclusion studies [5]. This region has volcanic soils and a tropical climate with high annual rainfall (1000–1500 mm y^-1^), a combination of factors that is highly conducive to plant growth; and, unlike many productive grasslands, fire is absent. Importantly, parts of eastern Java maintain relatively intact assemblages and densities of native herbivores [27, 28], unlike many other humid savannas globally that have been defaunated [29, 30]. In Malaysia, clearings adjacent to forests can boost ungulate numbers by providing a forage subsidy (i.e., access to crops [31]); whether analogous cross-boundary effects occur in Java remains unknown. In sum, Javan savannas have a unique combination of characteristics that make them a useful point of comparison to savannas elsewhere.

In Alas Purwo National Park in Java, Indonesia, we excluded large mammalian herbivores from ten 5 x 5 m plots for six months to experimentally assess how shifts in the herbaceous plant community along the growing season were modulated by herbivory. We tested the following hypotheses: (*i*) in the early growing season there would be no difference in plant species richness, evenness, or composition between plots exposed to herbivores (+LMH) or protected from herbivores (-LMH), (*ii*) by the end of the growing season, plots exposed to herbivores (+LMH) would have higher plant richness, lower biomass per quadrat, higher evenness, and their composition would differ strongly from exclosure plots (-LMH), (*iii*) the plant species composition of -LMH plots would diverge from each other across the growing season due to the combination of high productivity at Alas Purwo, the short duration of our experiment, and associated priority effects (e.g., [32]), (*iv*) the effects of large mammalian herbivores would be dependent on plant species identity and growth form, with grazing-intolerant forms benefitting most from herbivore exclusion.

## Materials and methods

### Study site: Historical aspects

The protected area that is now Alas Purwo National Park was originally designated in 1920 to protect 420 km^2^ of primary forest on the Blambangan peninsula. In 1939, conservationist F.J. Appelman lobbied for the inclusion of an adjacent 200 km^2^ tract of grassy wilderness that was renowned as one of the richest wildlife areas of Java [33]. While this extension was added to the protected area in the same year, it was degazetted in 1954 and planted with teak. The then ongoing destruction of this lowland savanna prompted the creation of artificial grazing grounds to provide grassland for the area’s large mammalian herbivores [34]. Between 1975–1979, Sadengan Feeding Ground (latitude, longitude: -8.65, 114.38) was cut out of the primary forest and seeded with native grasses from neighboring Baluran National Park [35]. Since then, Sadengan has been managed for large herbivores: park managers suppress fire, maintain artificial waterpoints, and mechanically control woody encroachment and non-native species where necessary. The complex nature of Sadengan—an artificial savanna carved out of natural forest created in response to the conversion of natural savanna into a timber plantation—provides a unique opportunity to study the ecological factors that maintain savannas.

### Study site: Biophysical aspects

**Fig 1:**
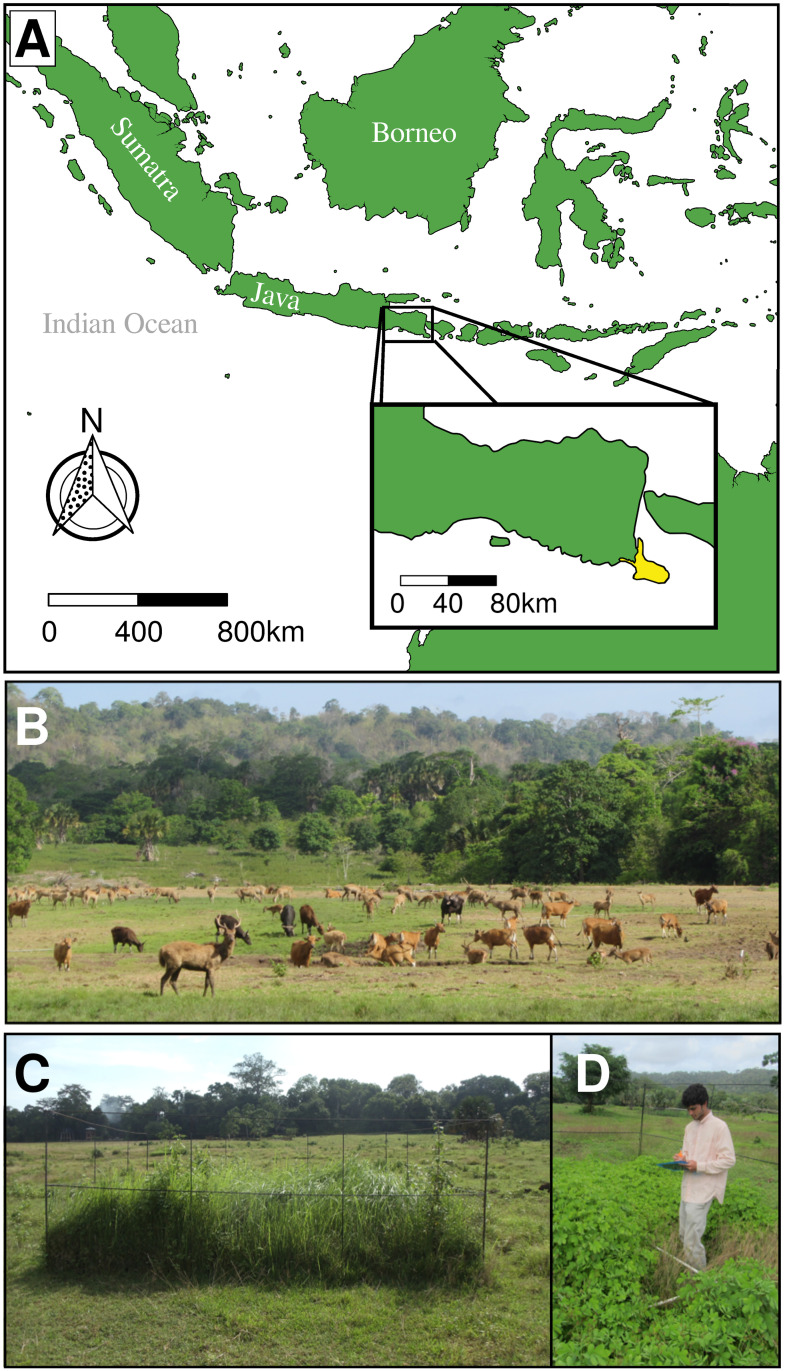
Overview of Sadengan Feeding Ground in Alas Purwo National Park. **A** Sadengan, the savanna habitat in Alas Purwo National Park (inset map; yellow area), is located in the south-eastern tip of Java, Indonesia. Map image was generated with the public domain global 10 m land shapefile from Natural Earth (naturalearthdata.com) and R. **B** Despite its small size (1 km^2^), experimental origin, and encapsulation by moist deciduous forest, Sadengan supports a dense ungulate fauna comprising four native ungulate species including the endangered banteng (*Bos javanicus*). Banteng and Javan rusa deer (*Rusa timorensis*) were the most common ungulates in our surveys (*n* = 4 surveys; mean individuals counted ± 1 SEM = 52.25 ± 16.93 and 62.75 ± 12.63, respectively) followed by banded pigs (*Sus scrofa vittatus*; 4.25 ± 1.44) and barking deer (*Muntiacus muntjak*; 0.25 ± 0.25). **C-D** Two of the exclosure plots from our study after five months of large herbivore exclusion, illustrating dominance achieved by *Imperata cylindica* and *Senna* cf. *tora*, respectively.

Sadengan’s grassland lies on a coastal plain approximately 1.5 km from the Indian Ocean and adjacent to the low limestone hills of the Blambangan peninsula (Fig 1). The soil was identified as a vertisol (black cotton soil); local farmers report that it is very fertile (*pers*. *comm*., to ABP) and soil tests corroborate this [36]. Alas Purwo National Park falls within the tropical wet and dry zone (Aw in the Köppen climate classification system). Total yearly rainfall is between 1000–1500 mm [37]. Fire is suppressed by park authorities. The dominant vegetation is moist deciduous forest with abundant bamboo; early successional elements are notably absent [38].

Sadengan has one of the few nearly intact faunal assemblages remaining in Java. Banteng (*Bos javanicus*; IUCN status: EN), rusa deer (*Rusa timorensis*; IUCN status: VU), banded pigs (*Sus scrofa vittatus*), and barking deer (*Muntiacus muntjac*) are all found on Sadengan’s grassland. Mouse-deer (*Tragulus javanicus*) are found in the forested areas of Alas Purwo. Of these, banteng and rusa are thought to be preferentially grazers [39, 40], banded pigs are omnivores, whereas barking deer and mouse-deer are browsers. The top predators are the dhole (*Cuon alpinus*; IUCN status: EN) and the Javan leopard (*Panthera pardus melas*; IUCN status: CR). Weedy plant species of management concern have encroached upon Sadengan; in particular, cogon grass *Imperata cylindrica*, forbs *Senna cf*. *tora* and *Chromolaena odorata*, as well as the tree *Senna siamea* [41], the last three of which are periodically controlled. Permission to undertake this study was kindly given by Kementerian Riset dan Teknologi Republik Indonesia (Indonesian Ministry of Research), Balai Konservasi Sumber Daya Alam Jawa Timur (Chamber of Natural Resource Conservation), and Taman Nasional Alas Purwo (Alas Purwo National Park). The individual identified in Fig 1D of this manuscript (ABP) has given written informed consent (as outlined in PLOS consent form) to publish their identifying information.

### Experimental design

We erected 10 grazing exclosures (5 x 5 m; 2 m tall) at Sadengan in November 2012; the start of the wet season. At the onset of the experiment, herbaceous canopy height was extremely low (less than 3 cm tall) and vegetation appeared homogeneous. The short, sprouting appearance of the vegetation indicated that the first rains had arrived only 2–3 weeks earlier.

Large-herbivore exclosures were placed haphazardly in the open grassland to avoid features such as trees, termite mounds, artificial waterpoints, and riverbeds. The southeastern quadrant of Sadengan was avoided, on account of its inaccessibility and the predominance of undesirable species, which were being manually cleared in that area at the time of the study. A paired 5 x 5 m control plot was placed two meters to the north of each exclosure plot.

Each grazing exclosure had a frame of welded steel bars: the bottom 1 m was covered with chain-link fence, while the top 1 m was covered with four strands of fencing wire, later interwoven with the thorny branches of a *Caesalpinia* sp. to deter inquisitive ungulates. This fencing excluded all ungulates but did not hinder the passage of small mammals, reptiles, and birds. The chain-link fence (mesh size = 6 cm x 6 cm) cast minimal shade and did not noticeably reduce wind speed. Birds seldom perched on the exclosures.

### Data collection

We recorded plant species composition based on percent cover in both the exclosures and the control plots at two time points: in early January 2013 (7 weeks after experiment initiation; early growing season) and again in April 2013 (24 weeks after experiment initiation; late growing season). We selected two 2 x 2 m subplots within each plot, with their center points 1 m to the north or south of the plot center point. Each 2 x 2 m subplot was subdivided into four 1 x 1 m quadrants. One of the two 2 x 2 m subplots was assigned to percent cover surveys (the other to biomass clipping to be undertaken at the end of the growing season; see below). Within each subplot assigned to percent cover surveys, we visually estimated the percent canopy coverage of each plant species (and bare ground) within each 1 x 1 m quadrant. Our methods are identical to those from similar studies [20, 21, 23, 42], except that canopy coverage was estimated to the nearest 1% for values less than 10% and to the nearest 5% for values between 10% and 100%. Species with estimated cover of less than 1% were recorded as 0.5%. Since the coverage of each species was estimated independently and canopies overlapped, total coverages often exceeded 100%. Coverage for each 2 x 2 m subplot was computed as the average coverage composition of each of the four constituent quadrants. Plant species were identified with the help of Flora of Java (1963–1968) [43] and through consultation with local experts.

In May and June (at the end of the growing season and ~28 weeks post experiment initiation), we clipped aboveground biomass in the 2 x 2 m subplot within each experimental plot (-LMH, +LMH) that had been reserved for biomass clipping. Clipped biomass was used to (*i*) provide a metric of aboveground biomass per quadrat in each experimental plot, and (*ii*) assess species composition based on biomass (as a complement to estimates based on percent cover). Standing biomass was clipped within each 1 x 1 m quadrant of the 2 x 2 m subplot: within each quadrant, we clipped a centrally located 0.2 m x 0.5 m frame (i.e., one tenth of the 1 x 1 m quadrat, or 0.1 m^2^). Plants were clipped at ground level or at the crown for perennial grasses. Only the portions of plants rooted within the frame were clipped. Living biomass was sorted to species level, while dead plants and unattached dead plant parts (of all species) were considered as litter. The samples were oven dried for two days at 60°C, which was sufficient to reach a constant weight. Summing live and litter portions of aboveground biomass yielded a proxy for aboveground standing crop in each plot (grams dry matter per 0.1 m^2^; weighed to nearest 0.01 gram, averaged across the four 0.2 x 0.5 m quadrats in each plot; hereafter “biomass per quadrat”) at the time of peak biomass, and did not capture the plant biomass in +LMH plots that had been consumed by large mammalian herbivores across the growing season. Living biomass was used to generate biomass-derived estimates of species composition as a complement to species composition analyses based on percent cover. Given the lower level of sampling for species composition based on biomass (both in spatial extent within plots and across seasons), we present data from percent cover surveys in the main text.

To verify that large-mammal herbivory was a major feature of Sadengan’s savanna, we counted large mammalian herbivores on the grassland from a watchtower at one end of the habitat. Counts were made on four occasions between October 2012 and June 2013 in morning or late afternoon (when herbivores are most active [28]) for a duration of approximately 1 hour and 20 minutes each.

### Statistical analyses

To test our hypotheses on the role of large mammalian herbivores in driving plant community development along the growing season at Sadengan, we used data on the plant species composition (as derived from both percent cover and biomass) and biomass per quadrat.

First, we computed broad metrics of plant community structure (species richness, evenness, and biomass per quadrat i.e., grams dry matter per 0.1 m^2^) to describe the effect of large mammalian herbivore exclusion on Sadengan’s plant community. Richness and evenness were computed from the percent-cover-derived species composition data for each plot and estimated for both the early season and late season. Evenness was Pielou’s evenness [i.e., Shannon diversity divided by *ln*(richness)]. Biomass per quadrat (grams dry matter per 0.1 m^2^) for each plot was the sum of live biomass and litter biomass within each 0.2 x 0.5 m frame. To estimate the effect of large mammalian herbivore exclusion, we calculated a standardized effect size as the log-response ratio [*ln*(+LMH/-LMH)] for each metric and each pair of -LMH and +LMH plots. A positive value indicated that the metric was greater where herbivores were present (+LMH) and a negative value indicated that the metric was greater in exclosures (-LMH). We used paired *t*-tests to assess the significance of experimental effects on each metric.

Next, we assessed the degree to which herbivore presence influenced plant community composition. For each of our measures of plant-species composition (early-season percent cover, late-season percent cover, and late-season percent biomass), we used partial distance-based redundancy analysis (dbRDA) to test whether herbivore presence predicted dissimilarity in plant community composition. Percent cover measures were first converted to relative abundance by dividing each percentage by the plot sum to ensure that relative cover in each plot summed to one. For each descriptor of species composition, we computed the compositional dissimilarity of plant communities between plots as the Bray-Curtis dissimilarity with the ‘vegdist’ function in the R package ‘vegan’ (v2.5.6) [44]. The matrix of compositional dissimilarities among plots was used as the response variable in partial dbRDA analysis, which was conditioned on experimental block to control for by-block differences and constrained by plot treatment (i.e., +LMH, -LMH). We used assessed model fit with adjusted *R*^2^ and quantified the importance of herbivore presence for among-plot dissimilarities with permutational ANOVA (*n* = 9999) using the ‘anova’ method in ‘vegan’ (v2.5.6).

To assess how herbivore presence altered the development of plant communities across the growing season, we also computed the change in percent cover of each plant in each plot between early and late season surveys. Using the same partial redundancy analysis (partial RDA) approach as above, we tested whether herbivory presence influenced plot-level patterns of plant growth and senescence across the growing season. In this case, the response variable of our partial RDA was the matrix of plot-by-plant changes in percent cover across the growing season. As before, partial RDA was conditioned on experimental block and constrained by plot treatment. We again used adjusted *R*^2^ to assess model fit and permutational ANOVA (*n* = 9999) to assess the contribution of plot treatment to variation in plant community composition.

Narrowing our focus to assess if herbivores impacted particular plants more than others, we then tested the hypothesis that growth form and species identity determined herbivore-driven impacts on plant cover, biomass per quadrat, and growth. To do so, we examined each plant species’ response to the loss of large mammalian herbivores using linear mixed-effect models. Plant species responses were estimated as the log-response ratio of late-season percent abundance (i.e., percent cover or percent biomass) between paired -LMH and +LMH plots [i.e., *ln*(+LMH/-LMH)]. For this analysis, we limited our dataset to those species recorded in both +LMH and -LMH plots within an experimental block during late-season surveys to ensure an effect could be computed. We assessed the importance of predictor variables (species identity, growth form) using likelihood-ratio tests that compared models including and excluding the variable of interest; we consider the variable of interest to be important if the model including that variable explained significantly more of the variation than the null at α = 0.05. In the first model we used plant species’ growth form as a fixed effect and experimental block and species identity as crossed random effects. In the second model, we used plant species’ identity as a fixed effect and experimental block as a random effect to test whether particular plant species responded to herbivory more strongly than others. In each case, the null model only included the random effects. Both models were implemented with the R package ‘glmmTMB’ (v0.2.3). To visualize seasonal growth trajectories for each plant species, we also computed the difference between each species’ percent cover within a given plot in the late season and early season (for the species that were observed in the late season). Because percent cover estimates received greater sampling effort (in some plots we were not able to generate complete biomass-based composition data and these plots were therefore excluded from analyses), we present results for species composition based on percent cover rather than biomass-derived species composition, which are presented in the Supplementary Material.

Throughout the Results, Tables, and Figures, we present the mean and one standard error of the mean to summarize each measure.

## Results

**Fig 2:**
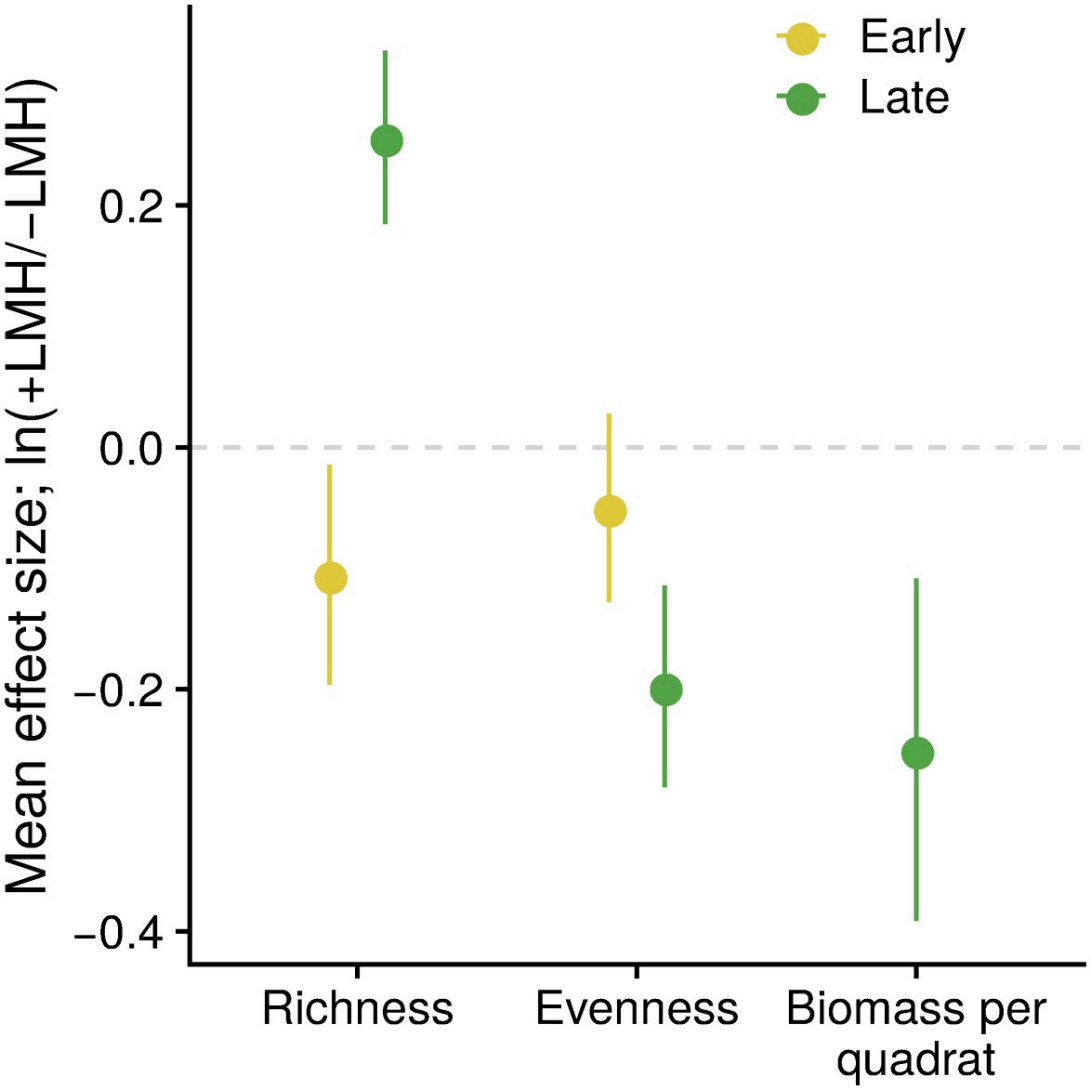
Response of plant communities to herbivory. On average, the presence of large mammalian herbivores led to an increase (as shown by the log-response ratio of control-exclosure comparisons, *y*-axis) in plant-community richness, a decrease in evenness, and a decrease in biomass per quadrat relative to exclosures by the end of the growing season. These differences were mostly not statistically significant. On average, species richness was lower in +LMH plots compared to -LMH plots in the early season and then greater in +LMH by the late season although neither effect was significant (paired *t*-tests: *t* = -1.38, *DF* = 9, *P* = 0.20 and *t* = 1.12, *DF* = 9, *P* = 0.29, respectively). Plant-community evenness tended to always be lower in +LMH plots but this was only significant in the late season (paired *t*-tests: *t* = -0.62, *DF* = 9, *P* = 0.55 and *t* = -2.35, *DF* = 9, *P* = 0.04, respectively). Biomass per quadrat (grams dry matter per 0.1 m^2^) at the end of the growing season was lower, on average, in +LMH plots but this was not significant (paired *t*-test: *t* = -1.75, *DF* = 9, *P* = 0.11).

**Fig 3:**
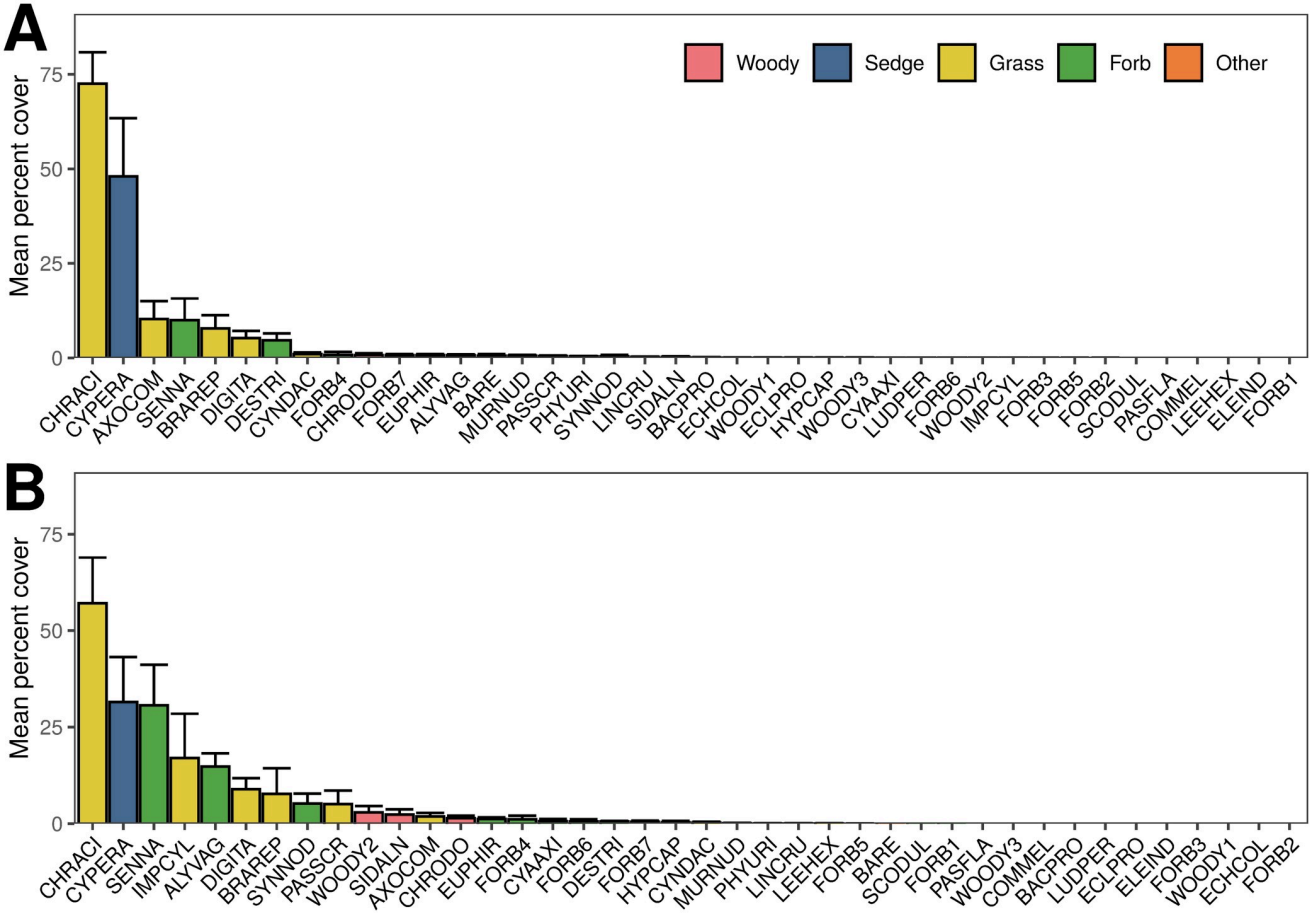
Distribution of plant species’ mean percent cover in the late growing season. **A-B** Percent-cover distributions describe the composition of Sadengan’s plant community in the late growing season in **A** control (+LMH) plots where large mammalian herbivory alters the plant community, and **B** exclosure (-LMH) plots where large mammalian herbivory is absent. In general, Sadengan’s plant community was dominated by graminoids and forbs with few woody plants (though these did represent greater percent cover in -LMH plots; **B**). The most common plant taxa (CHRACI, *Chrysopogon aciculatus* and CYPERA, Cyperaceae sp.) were dominant in both plot types, however the magnitude of their dominance was lower where large mammalian herbivores were excluded (**B**). Three taxa in particular (SENNA, *Senna* cf. *tora*; IMPCYL, *Imperata cylindrica*; ALYVAG, *Alysicarpus vaginalis*) increased in percent cover as a result of decreased dominance of CHRACI and CYPERA. The complete list of codes used in these figures and their taxonomic names are in S1 Table. Percentages do not sum to 100% due to canopy overlap.

In general, Sadengan’s herbaceous plant community was highly heterogeneous. By the late growing season, plots exposed to herbivory typically comprised 15 species per 2m^2^ subplot (15.2 ± 1.14) despite 33 species being recorded across the ten plots. Plant-species richness was slightly lower in +LMH plots in the early season and higher in +LMH in the late season but these differences were not significant (Fig 2). Plant-species evenness was significantly lower in +LMH plots by the late growing season but did not differ between plots in the early season (Fig 2). Biomass per quadrat tended to be lower in +LMH plots by the end of the growing season but not significantly so (Fig 2). Of the 39 taxa cumulatively recorded across all plots and seasons (Fig 3), more than half were forbs (54%), grasses accounted for another quarter (28%), and woody plants accounted for much of the rest (15%). Sedges were lumped into a single taxon to avoid taxonomic ambiguities. At the end of the growing season, plots with large mammalian herbivores (+LMH) were dominated by grasses and sedges (percent cover, 97.4% ± 3.4% and 48.0% ± 15.4% cover, respectively) whereas forbs and woody plants were sparse (combined percent cover, 20.8% ± 7.0%; Fig 3A). However, plots without large mammalian herbivores (-LMH) had greater forb and woody-plant cover (62.8% ± 10.4%; Fig 3B).

**Fig 4:**
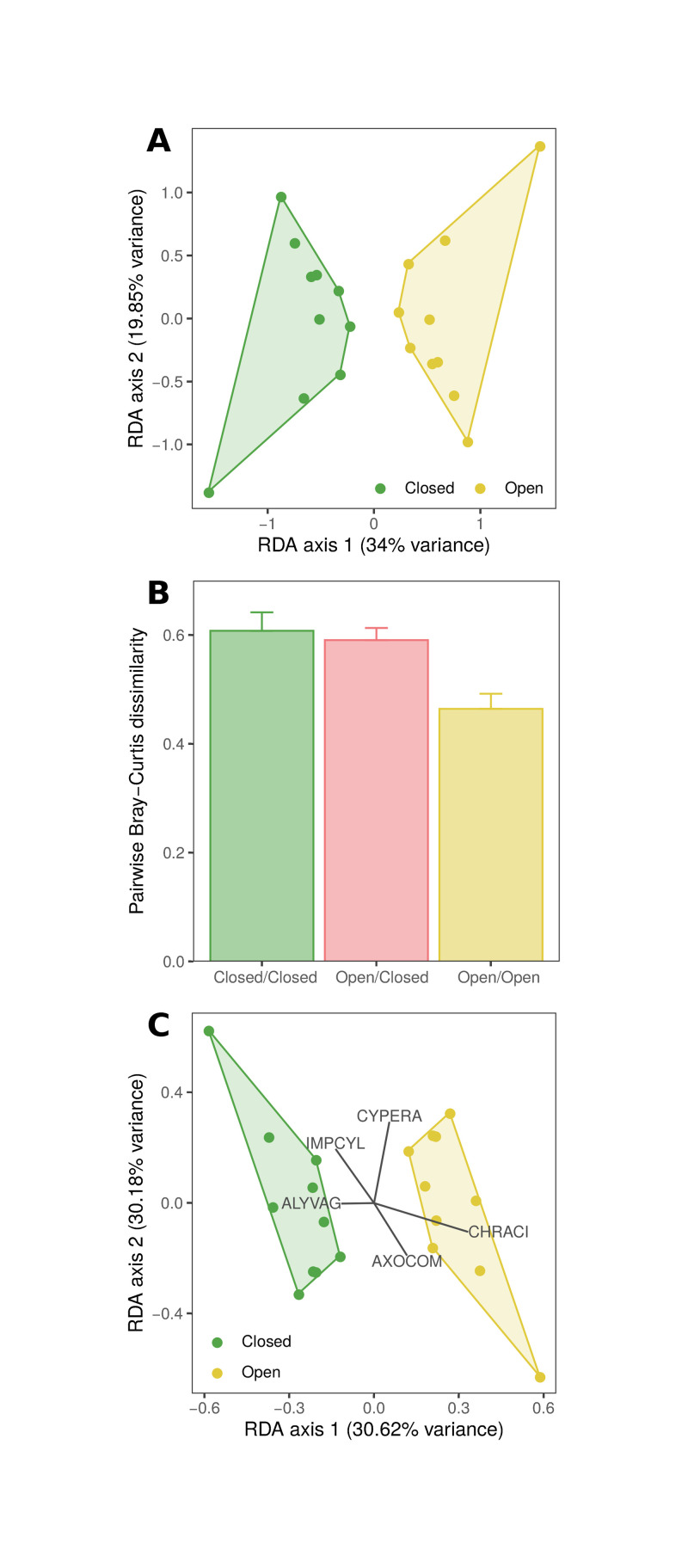
Plant species composition varied with herbivory presence. **A** Partial distance-based redundancy analysis (dbRDA) of late season plant-community composition (percent cover; Bray-Curtis dissimilarity) conditioned on experimental block and constrained by plot treatment identified a significant contribution of herbivore presence (i.e., plot treatment; represented by *x*-axis) to compositional dissimilarity between plots (adjusted *R*^2^ = 0.22; permutational ANOVA, *n* = 9999; *F*_1,9_ = 4.64, *P* = 0.004). **B** On average, pairwise Bray-Curtis dissimilarities were greatest between Closed plots (-LMH; 0.61 ± 0.03) and smallest between Open plots (+LMH; 0.46 ± 0.03) indicative of a homogenizing effect of large mammalian herbivores on the plant community assembly across the growing season. **C** Partial redundancy analysis (RDA) of seasonal changes in species’ percent cover within each plot (i.e., the difference between late- and early-season percent cover) conditioned on experimental block and constrained by herbivore presence (i.e., plot treatment) attributed a significant effect of herbivory (*x*-axis) to changes across the growing season in the percent cover of plant species in each plot (adjusted *R*^2^ = 0.18; permutational ANOVA, *n* = 9999; *F*_1,9_ = 3.97, *P* = 0.004). Species vectors are shown for the five species with the largest dispersions from the center. Specifically, the growth of *Imperata cylindrica* (IMPCYL) and *Alysicarpus vaginalis* (ALYVAG) was closely associated with -LMH plots whereas *Chrysopogon aciculatus* (CHRACI) and *Axonopus compressus* (AXOCOM) growth was associated with +LMH plots; Cyperaceae sp. (CYPERA) growth was associated with unidentified variation along the *y-*axis.

Herbivore presence had a significant impact on plant community composition (as estimated by percent cover of plant taxa) over the course of the growing season at Sadengan. Partial redundancy analysis (partial dbRDA) of late growing-season compositional dissimilarity identified a significant effect of herbivore presence on plant community composition (Fig 4A). This effect emerged across the growing season because differences in early growing-season composition were not strongly associated with herbivore presence (S1 Fig). Pairwise dissimilarity of species composition in the late growing season was greatest between plots where herbivores were excluded (-LMH; 0.61 ± 0.03) and smallest between plots exposed to herbivory (+LMH; 0.46 ± 0.03) suggesting that herbivory constrained plant community development along the growing season (Fig 4B). These results were qualitatively similar, although the differences were less pronounced, when assessed using the biomass of each plant taxon (per 0.1 m^2^) at the end of the growing season (S2 Fig).

Herbivore presence also impacted the growth trajectories of plant species across the growing season. We found a significant effect of herbivore presence on the changes in species-wise percent cover across the growing season. Partial redundancy analysis of the change in each plant species’ cover in each plot between the early and late growing season identified a significant effect of plot treatment (Fig 4C).

**Fig 5:**
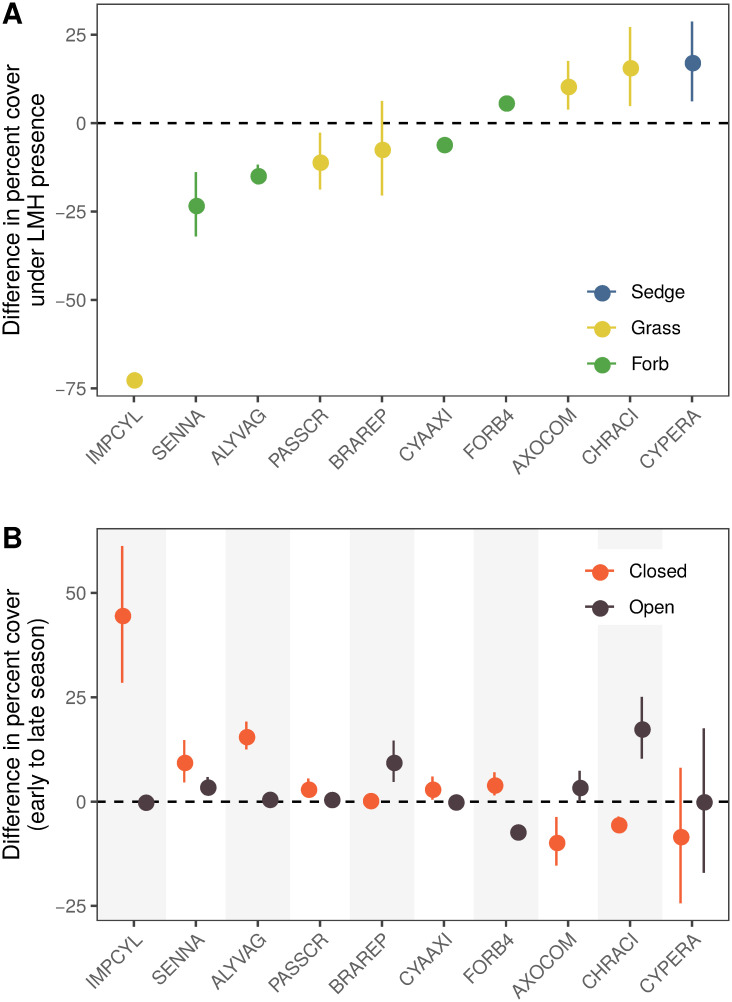
Impacts of herbivory on specific plant species varied. Plant species are referred to by six-letter codes with corresponding identities in S1 Table. **A** The mean difference in late season percent cover (± 1 SEM) in +LMH plots compared to -LMH plots is shown for the ten species that responded most strongly to exclusion (results for all species for which an effect could be calculated are shown in S3 Fig). A small set of taxa, which were mainly graminoids, increased in cover where herbivores were present: sedges (CYPERA), *Chrysopogon aciculatus* (CHRACI), and *Axonopus compressus* (AXOCOM), whereas two forbs (SENNA, *Senna* cf. *tora*; ALYVAG, *Alysicarpus vaginalis*) and the aggressively growing grass, *Imperata cylindrica* (IMPCYL), had appreciably smaller late season cover where herbivores were present compared to exclosures. **B** Herbivore presence also modified the seasonal growth trajectories of some plants. The *y-*axis shows the change in percent cover of the same ten plant species as in **A** between the early and late season and segregated by herbivory presence. The ‘winners’ of herbivore exclusion (at left in **A**) grew more where herbivores were excluded (‘Closed’, -LMH) compared to where herbivores were present (‘Open’, +LMH; at right in **B**). Plant taxa that increased in cover where herbivores were present (at right in **A**), also showed varied seasonal growth patterns based on herbivore presence. Sedges (CYPERA) generally maintained cover throughout the season but decreased in some herbivore exclusion (‘Closed’, -LMH) plots possibly due to stronger competition there. Likewise, the grasses CHRACI (*Chrysopogon aciculatus*) and AXOCOM (*Axonopus compressus*) decreased in percent cover across the growing season where herbivores were excluded, in contrast to their increased percent cover over the growing season where herbivores were present.

The effects of large mammalian herbivores appeared to be skewed towards particular plant species but were inconsistent within growth forms. Growth form (i.e., forb, grass, sedge, woody) was not a significant predictor of variation in plant responses to herbivore exclusion (likelihood-ratio test between a model that included a predictor for growth form and one without: χ^2^ = 1.90, DF = 4, *P* = 0.75). Instead, plant species’ identity significantly predicted plant responses to herbivore exclusion (likelihood-ratio test between a model that included species identity as a predictor and one without: χ^2^ = 77.68, DF = 22, *P* < 0.001). In particular, the graminoid taxa that dominated plant communities in the presence of herbivores (sedges, CYPERA; *Chrysopogon aciculatus*, CHRACI) were more abundant where herbivores were present compared to where herbivores were excluded (Fig 5A and S3 Fig) whereas herbaceous species including the invasive legume *Senna* cf. *tora* (SENNA) and an aggressively growing grass (*Imperata cylindrica*; IMPCYL) were less abundant where large-herbivores were present compared to exclosures (Fig 5A). Species that ‘won’ from herbivore exclusion grew more during the growing season where herbivores were absent compared to where they are present (at left in Fig 5B and S3 Fig). Species that ‘lost’ from herbivore exclusion declined in abundance across the growing season where herbivores were absent compared to increasing through the season where herbivores were present (e.g., CHRACI and AXOCOM in Fig 5B).

## Discussion

The development of herbaceous plant communities across the growing season on Sadengan’s savanna was strongly modified by the presence of large mammalian herbivores. As hypothesized, there was little difference in plant community composition, richness, or evenness in the early growing season. However, differences in composition did emerge over the growing season. Where herbivores were present, plant communities were more similar, richness was higher (though non-significantly so), and evenness was lower; this was contrary to our expectation that large mammalian herbivores would promote evenness. Evenness increased under large mammalian herbivore exclusion, likely due to declines in dominant graminoid taxa and increases in the weedy *Imperata cylindrica* grass and invasive forb *Senna* cf. *tora*. Directional shifts in plant community composition and the greater compositional variation by the end of the growing season resulting from herbivore removal suggest that herbivory limited the spectrum of plant community compositions. Herbivores likely manifest these impacts via a combination of selective foraging, which modulates light competition, and physical impacts such as trampling. While our experiment did not tease apart these mechanisms, we will discuss them briefly here.

The short-term nature of our experiment offers a valuable comparison to other herbivore-exclusion experiments. By focusing on just one growing season, we were able to show that, beginning from a denuded landscape at the onset of the experiment where vegetation was only a couple of centimeters high, distinct community types emerged within a six-month period according to herbivore presence, which accords with compositional shifts seen in other productive sites [19]. As found in other herbivore-exclusion experiments [21] and global syntheses of such experiments [5], we observed clear compositional changes but variation in simpler metrics such as species richness was less evident.

Concordant with the idea that herbivory structures the baseline plant community of Sadengan’s grassland, there was evidence of heavy utilization by large mammalian herbivores. The dominance of *Chrysopogon aciculatus*, a prostrate grass, which is considered alongside *Axonopus* and *Desmodium* as an indicator of heavy stocking rates [45], suggests that Sadengan possesses a grazing-tolerant plant community. These species (as well as *Brachiaria* and *Cynodon*) have a prostrate growth habit, a trait associated with resistance to trampling [46] and herbivory tolerance [47], and tended to decline within grazing exclosures. In contrast, the species that tended to increase in exclosures tended to be more upright-growing species (such as *Senna* cf. *tora*) that better compete in savannas when light is limiting [48]. This parallels results in Pangandaran reserve (West Java, Indonesia), where heavily grazed areas were dominated by prostrate grasses *Axonopus*, *Cynodon*, and *Paspalum conjugatum*, and plots with the lowest grazing intensity were often dominated by the upright *Imperata cylindrica* [49]. As such, the general idea that fertile, wet grasslands should exhibit strong divergence into either grazing tolerant and canopy dominant communities depending on grazing intensity [6, 18] was generally supported.

The responses to herbivore exclusion that we observed were dominated by three species: *Chrysopogon aciculatus*, *Senna cf*. *tora*, and *Imperata cylindrica*. *Chrysopogon* dominated herbivore-exposed areas and declined without herbivores, but the magnitude of this effect was variable. When *Chrysopogon* declined heavily it was replaced by either an invasive forb (*Senna* cf. *tora*) or aggressive grass (*Imperata cylindrica*). The occasional takeover by these species inside exclosures implies that they are otherwise effectively controlled by large mammalian herbivores on Sadengan. *Imperata cylindrica* is considered one of the most difficult-to-control plant species in the world, as it is thought to be low value for livestock [50, 51] and is both fire tolerant and highly flammable. As such, it creates fire-dominated monocultures [52] covering an estimated 35 million hectares in Asia [53]. However, multiple studies support our result that wild ungulates effectively control *Imperata cylindrica*; it is preferential forage in India and Nepal [54–57], as well as in Uganda [58]. This strongly suggests that selective foraging, demonstrated elsewhere to effectively control plant species of management concern [12], suppresses *Imperata cylindrica*.

The increase in *Senna* cf. *tora* in exclosures was contrary to our hypothesis that selective foraging should lead to higher abundance of unpalatable species in herbivore-exposed plots. *Senna tora* and the very similar and closely related *Senna obtusifolia* are both unpalatable neotropical weeds of pastures [59, 60]; the invasion of *Senna* cf. *tora* is considered to have greatly reduced the grazing value of Sadengan for banteng, which avoid it [41]. *Senna obtusifolia* has been investigated for its deer-repellent properties [61], and the *Senna* cf. *tora* in this study anecdotally showed very little browse damage, suggesting that it may also be toxic. *Senna* cf. *tora* increased across the growing season at a faster rate in the herbivore-exclusion plots when compared with the herbivore-exposed controls, suggesting that native herbivores may be slowing its spread. Erect forbs have particularly low resistance to trampling [46], suggesting that one way that large mammalian herbivores might limit the growth of this unpalatable weed.

Because much of insular Southeast Asia is forested, it is often forgotten that savannas do occur in the region [62], and little is known about their maintenance and even which areas are natural [63] and which are anthropogenic [52, 53, 64]. We show that Sadengan’s savanna, although artificially created, exhibited many of the ecological characteristics of natural savannas. The fact that the savannas of Java are grazed by a mostly intact assemblage of wild bovids and cervids is significant, and may indicate that increased access to preferred foraging resources can facilitate ungulates that are otherwise found at low densities in forested habitats [31, 65]. Furthermore, this initial set of evidence suggests that large mammalian herbivores contribute to Sadengan’s maintenance as a functional savanna, constraining plant community composition within the growing season and promoting grazing-tolerant, prostrate plant species, whose cover was much reduced within exclosures. In addition, the observation that Sadengan’s herbivores contribute to the control of two undesirable, weedy plant species underlines the importance of this large mammalian herbivore assemblage for the maintenance of Sadengan as a savanna. Importantly, Sadengan’s savanna provides critical habitat for threatened bird species, including Green Peafowl (*Pavo muticus*; IUCN status: EN), Lesser Adjutant (*Leptoptilos javanicus*, IUCN status: VU), Black-winged Myna (*Acridotheres melanopterus*, IUCN status: CR), and occasionally Javan Hawk-Eagle (*Nisaetus bartelsi*, IUCN status: EN) [66], the first three of which have been observed associating with grazing animals in Sadengan. Globally, savannas are a threatened, declining biome [67]. Restoring savannas, rewilding their biotic assemblages [1], and abiotic regimes, particularly in areas where they can easily be lost to forest or agriculture, is therefore a critical part of global biodiversity conservation actions [68].

## Supporting information

S1 Fig Compositional differences were low in the early-growing season. **A** Partial distance-based redundancy analysis (dbRDA; Bray-Curtis dissimilarity) based on early season percent cover of plants in experimental plots. Plot treatment (represented by the *x-*axis) was a non-significant predictor of dissimilarity in plant-community composition (dbRDA conditioned on plot identity; adjusted *R*^2^ = 0.06; permutational ANOVA, *n* = 9999; *F*_1,9_ = 1.67, *P* = 0.15). While the *x*-axis represents plot treatment and separates the plant communities, it is a poor representation of variation in plant communities as it only accounted for 16% of variation in plot composition whereas the first three unconstrained axes, representing unaccounted variation, together account for close to 70%. **B** Pairwise Bray-Curtis dissimilarity of plots did however suggest treatment effects were beginning to emerge after six weeks of herbivore-exclusion. Plots where herbivores were absent (Closed plots) were more dissimilar to each other (mean ± SEM: 0.50 ± 0.03) than were plots where large mammalian herbivores were present (Open plots; mean ± SEM: 0.43 ± 0.02).
(EPS)

S2 Fig Compositional dissimilarity of plant communities based on plant species’ biomass. **A** Partial distance-based redundancy analysis (dbRDA) of plant community composition (based on species’ biomass; grams dry matter per 0.1 m^2^; Bray-Curtis dissimilarity) in the late growing season that was conditioned on experimental block and constrained by plot treatment (*x*-axis) revealed that plot treatment explained 25% of variation in plant community composition although it was not a significant descriptor (adjusted *R*^2^ = 0.13; permutational ANOVA, *n* = 9999; *F*_1,6_ = 2.04, *P* = 0.12). **B** Plant communities that were exposed to large mammalian herbivores (Open plots; +LMH) were most similar (pairwise Bray-Curtis dissimilarity; mean ± SEM: 0.57 ± 0.04) whereas plots where large mammalian herbivore species were removed (Closed plots; -LMH) were more dissimilar to each other (mean ± SEM: 0.71 ± 0.06).
(TIF)

S3 Fig Complete set of species-level effects of large mammalian herbivore exclusion. Difference in percent cover for the species found in at least one -LMH/+LMH pair during late season percent cover surveys. Species highlighted in Fig 3 are on the left- and right-most parts of each panel and are the most strongly responding species in the plots. Most plant species were rare and showed small absolute changes based on both season and herbivory.
(TIF)

S4 Fig Species-level changes in biomass due to large mammalian herbivore exclusion. Mean change in percent biomass (grams dry matter per 0.1 m^2^) of each plant species between +LMH plots and -LMH plots. Positive values indicate a species’ proportional biomass was greater in +LMH plots. As for percent cover, the presence of LMH led to decreases in the invasive forb *Senna* cf. *tora* (SENNA). Decreases in the dominant graminoids, which were observed in percent cover data, were also retrieved. Biomass data suggested larger increases in *Desmodium trifolium* (DESTRI) and larger declines in *Digitaria* sp. 1 (DIGITA) than percent cover data. Overall, species-level effects based on biomass data were less reliable than those based on percent cover; for biomass only 14 species were widespread enough (i.e., present in both plots of at least one experimental block) to estimate an experimental effect and, on average, when effects could be computed they were based on a mean of only 2.5 experimental blocks per species. Conversely, experimental effects from percent cover data could be computed for 23 species based on a mean of 4.3 experimental blocks per species.
(TIF)

S1 Table Key to plant species’ six-letter codes and taxonomic names. Codes used for each plant species in the study and corresponding finest taxonomic classification. Authorities given where relevant.
(TIF)
