# Supplementary figures and images for: Short-term plant-community responses to large mammalian herbivore exclusion in a rewilded Javan savanna
Source: PLoS One. 2021 Jul 22;16(7):e0255056. doi: 10.1371/journal.pone.0255056 (PMC8297766; doi:10.1371/journal.pone.0255056)

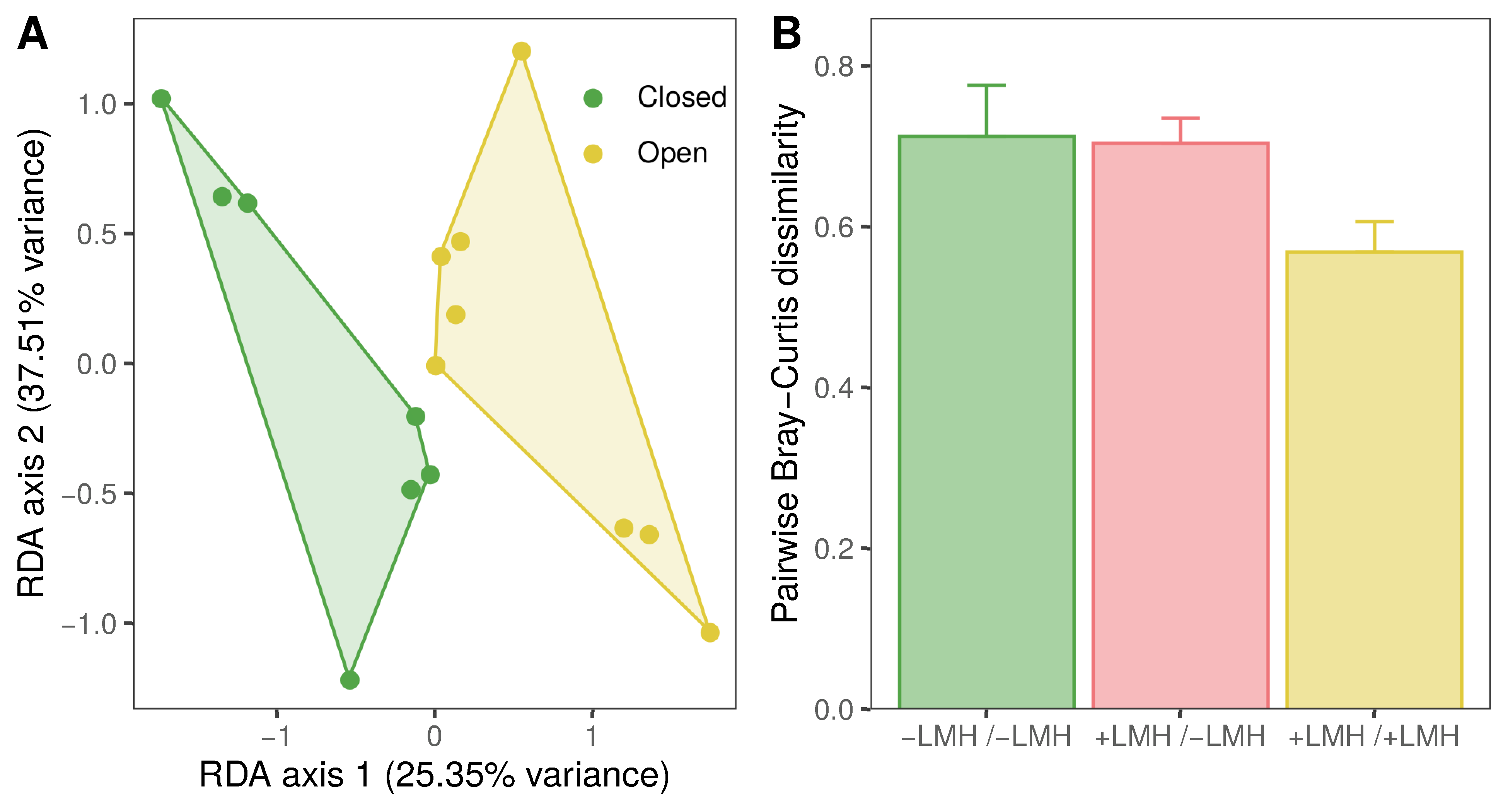

Supplement: S2 Fig — A Partial distance-based redundancy analysis (dbRDA) of plant community composition (based on species’ biomass; grams dry matter per 0.1 m2; Bray-Curtis dissimilarity) in the late growing season that was conditioned on experimental block and constrained by plot treatment (x-axis) revealed that plot treatment explained 25% of variation in plant community composition although it was not a significant descriptor (adjusted R2 = 0.13; permutational ANOVA, n = 9999; F1,6 = 2.04, P = 0.12). B Plant communities that were exposed to large mammalian herbivores (Open plots; +LMH) were most similar (pairwise Bray-Curtis dissimilarity; mean ± SEM: 0.57 ± 0.04) whereas plots where large mammalian herbivore species were removed (Closed plots; -LMH) were more dissimilar to each other (mean ± SEM: 0.71 ± 0.06). (TIF) [file pone.0255056.s002.tif]

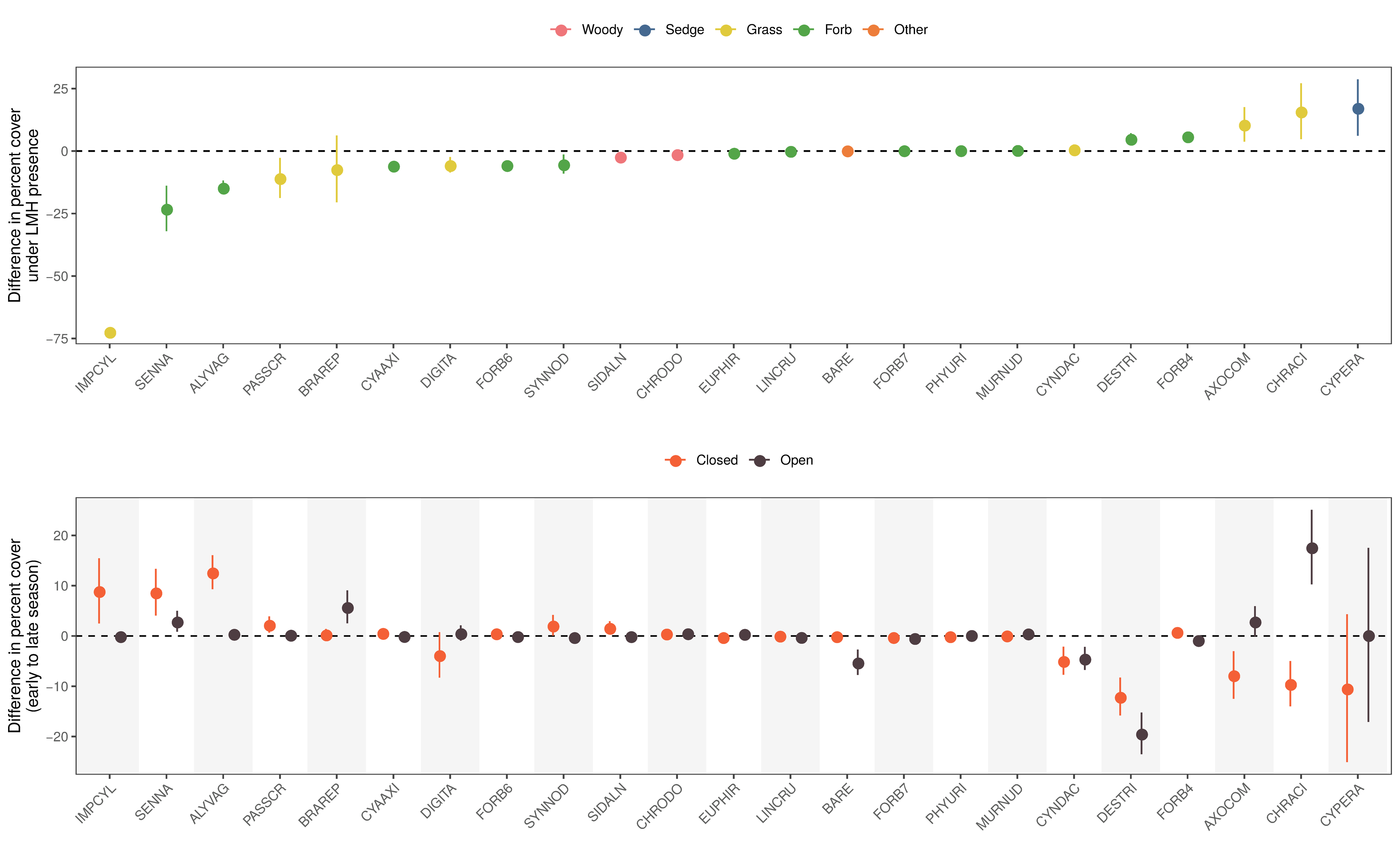

Supplement: S3 Fig — Difference in percent cover for the species found in at least one -LMH/+LMH pair during late season percent cover surveys. Species highlighted in Fig 3 are on the left- and right-most parts of each panel and are the most strongly responding species in the plots. Most plant species were rare and showed small absolute changes based on both season and herbivory. (TIF) [file pone.0255056.s003.tif]

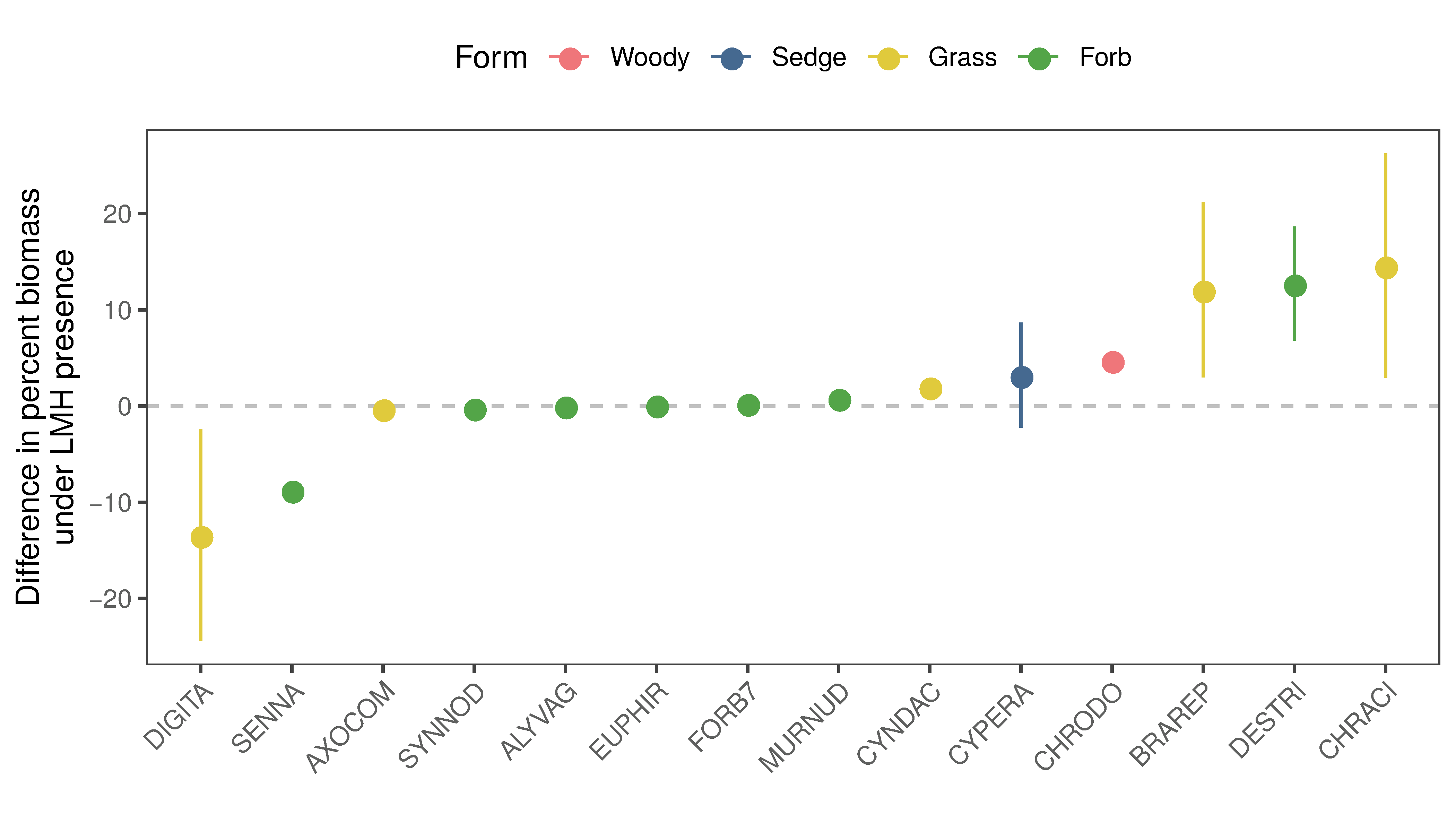

Supplement: S4 Fig — Mean change in percent biomass (grams dry matter per 0.1 m2) of each plant species between +LMH plots and -LMH plots. Positive values indicate a species’ proportional biomass was greater in +LMH plots. As for percent cover, the presence of LMH led to decreases in the invasive forb Senna cf. tora (SENNA). Decreases in the dominant graminoids, which were observed in percent cover data, were also retrieved. Biomass data suggested larger increases in Desmodium trifolium (DESTRI) and larger declines in Digitaria sp. 1 (DIGITA) than percent cover data. Overall, species-level effects based on biomass data were less reliable than those based on percent cover; for biomass only 14 species were widespread enough (i.e., present in both plots of at least one experimental block) to estimate an experimental effect and, on average, when effects could be computed they were based on a mean of only 2.5 experimental blocks per species. Conversely, experimental effects from percent cover data could be computed for 23 species based on a mean of 4.3 experimental blocks per species. (TIF) [file pone.0255056.s004.tif]

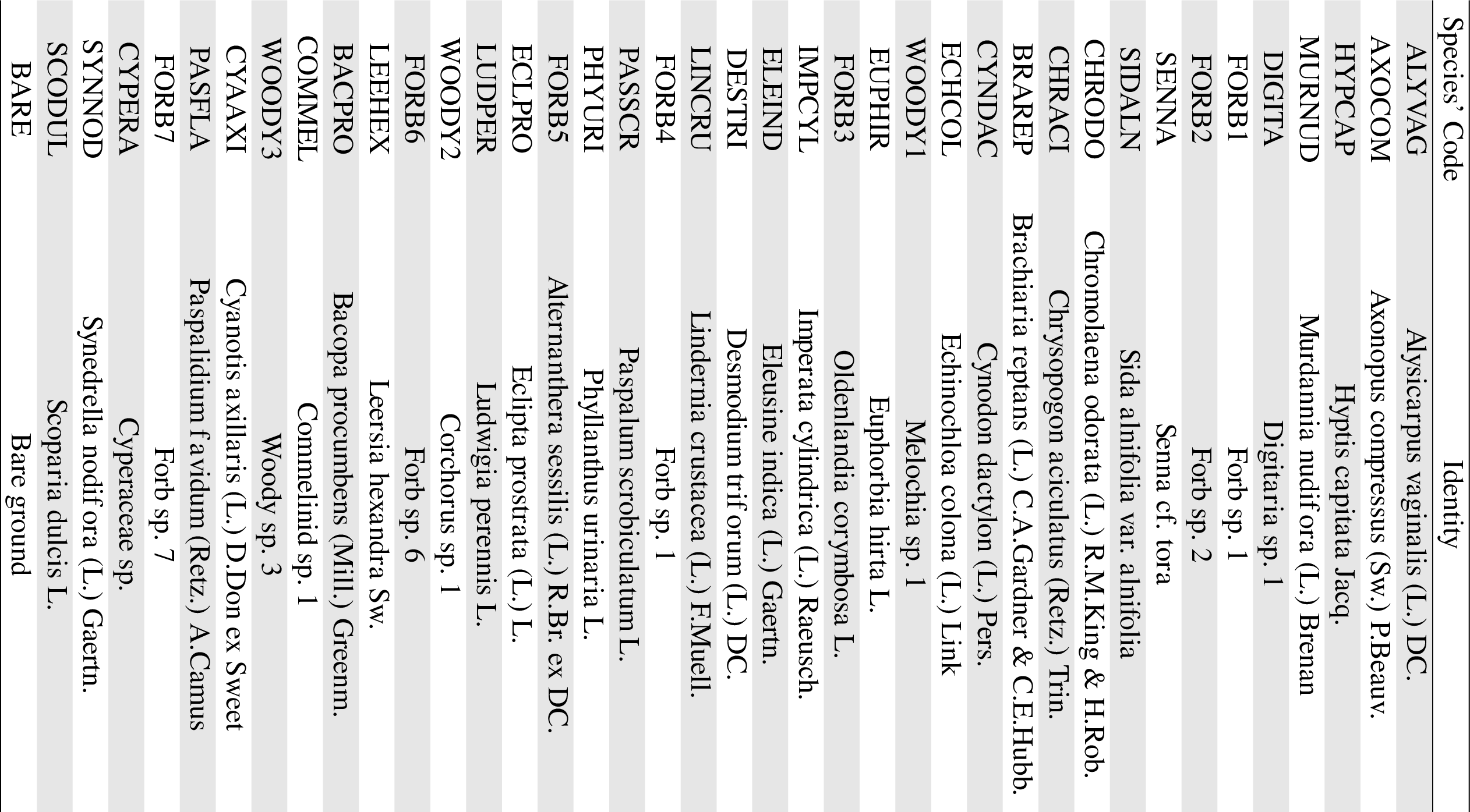

Supplement: S1 Table — Codes used for each plant species in the study and corresponding finest taxonomic classification. Authorities given where relevant. (TIF) [file pone.0255056.s005.tif]
